# Human Papillomavirus Vaccine Introduction in South Africa: Implementation Lessons From an Evaluation of the National School-Based Vaccination Campaign

**DOI:** 10.9745/GHSP-D-18-00090

**Published:** 2018-10-03

**Authors:** Sinead Delany-Moretlwe, Karen F. Kelley, Shamagonam James, Fiona Scorgie, Hasina Subedar, Nonhlanhla R Dlamini, Yogan Pillay, Nicolette Naidoo, Admire Chikandiwa, Helen Rees

**Affiliations:** a Wits Reproductive Health and HIV Institute (Wits RHI), Faculty of Health Sciences, University of Witwatersrand, Johannesburg, South Africa.; b National Department of Health, Pretoria, South Africa.

## Abstract

**Background:**

In April 2014, a national school-based human papillomavirus (HPV) vaccination program was rolled out in South Africa, targeting Grade 4 girls aged ≥9 years. A bivalent HPV vaccine with a 2-dose (6 months apart) schedule was used. At the request of the National Department of Health (NDoH), we conducted an external assessment of the first-dose phase of the vaccination program to evaluate program coverage and vaccine safety and identify factors that influenced implementation.

**Methods:**

We based our cross-sectional and mixed-methods approach on a process evaluation framework, which included a review of key planning and implementation documents and monitoring data; observation at vaccination sites; key informant interviews (N=34); and an assessment of media coverage and content related to the campaign.

**Findings:** There was overall success in key measures of coverage and safety. Over 350,000 Grade 4 girls were vaccinated in more than 16,000 public schools across South Africa, which translated to 94.6% of schools reached and 86.6% of age-eligible learners vaccinated. No major adverse events following immunization were detected. We attributed the campaign's successes to careful planning and coordination and strong leadership from the NDoH. The primary challenges we identified were related to obtaining informed consent, vulnerabilities in cold chain capacity, and onsite management of minor adverse events. While campaign planners anticipated and prepared for some negative media coverage, they did not expect the use of social media for spreading misinformation about HPV vaccination.

**Conclusions:**

The first phase of the national school-based HPV vaccination campaign was successfully implemented at scale in this setting. Future implementation will require improvement in the storage and monitoring of vaccine doses, better communication of role expectations to all stakeholders, and streamlined consent processes to ensure program sustainability.

## Abstract (precis)

Evaluation of the campaign confirmed its feasibility in this setting: it achieved high coverage, few adverse events, and mostly positive media coverage. However, challenges occurred in data and cold chain management. Future implementation requires improved partnerships between government ministries, simplified informed consent, and closer monitoring of social media messaging.

## INTRODUCTION

Incidence of cervical cancer in southern, central, and east Africa is among the highest in the world, and, despite being a preventable disease, it remains a leading cause of cancer mortality for women in these regions.^1^ In South Africa, 1 in 26 women develop cervical cancer during their lifetime,^2^ with most cases of invasive carcinoma present late, resulting in high fatality rates.^3^ Endemic levels of HIV infection among young women in the country are a strong contributing factor to this picture. HIV-infected women have a high prevalence of co-infection with human papillomavirus (HPV)^4^^,5^—the sexually transmitted virus responsible for almost all cases (99%) of cervical cancer—and tend to experience a poorer prognosis than women without HIV.^6^^,^^7^ Despite policy changes to improve coverage, screening uptake in South Africa is generally low,^8^ and there is high loss to follow-up of women identified with abnormal cytology.^7^

Traditional cytology-based screening procedures are likely to be replaced soon by more sensitive HPV testing,^9^ but a national HPV vaccination program is a critical component of effective *primary* prevention. Vaccinating girls prior to sexual debut (9 to 13 years), as recommended by the World Health Organization (WHO),^10^ is the most cost-effective public health measure against cervical cancer in high-prevalence settings.^11^

National HPV vaccination programs are a critical component of effective primary prevention of cervical cancer.

Evidence to inform public-sector introduction of HPV vaccination has emerged from recent evaluations of pilot projects and national programs in low- and middle-income countries.^12^^–^^16^ These evaluations show that coverage tends to be highest when vaccination is delivered through school-based programs, as found in settings as diverse as Australia,^17^ Bhutan,^18^ Peru,^19^ and the United States.^20^ However, with the introduction of any new vaccine, and despite good preparation, challenges often occur during the first year.^11^ These challenges may include service delivery weaknesses as well as concerns among communities and health workers about the relative newness of the vaccine, vaccine safety and side effects, and even the specific targeting of young girls.

In South Africa, early feasibility and acceptability studies^21^^–^^25^ and demonstration projects^15^ identified several potential areas of concern for implementing school-based HPV vaccination programs. The first set of concerns focused on human resource shortages, limited expertise with the delivery of a large-scale vaccination program, and the vaccination of pre-adolescents in general. In practical terms, capacity limitations can negatively impact every aspect of a national program, from gaining informed consent to the management of cold chain integrity.^21^

A second set of concerns arose in relation to potential opposition to an HPV vaccination program. In the context of a broader discourse about “sexual risk,” the HPV vaccine has acquired particular scientific and sexual meanings in every phase of its development, from discovery to distribution, marketing, and absorption into public health care systems around the world.^26^ As a result, the vaccine has become vulnerable to lobbying by diverse anti-vaccination and “vaccine-hesitant”^27^ advocacy groups. Feasibility and acceptability research to discover how receptive the public would be to vaccine messaging, undertaken prior to 2014, found strong support for HPV vaccination of young people among policy and health service representatives,^25^ parents, youth, and educators.^15^^,^^21^^,^^23^

In general, vaccines as a technology are widely accepted in South Africa, owing to familiarity with childhood vaccinations,^25^ which may partly account for these early indicators of support for HPV vaccination. While active opposition to the vaccine was not anticipated, policy makers did expect that some sectors of society might reject the HPV vaccine if the link to a sexually transmitted infection (STI) was too explicit.^25^ Similar concerns have arisen in other countries,^28^ and in South Africa fears about risk compensation and sexual permissiveness have surfaced as a popular response to condom provision and other sexual and reproductive health services in schools.^29^ To preempt possible opposition to HPV vaccination, policy experts advised a strategy of marketing the vaccine as preventing cervical cancer rather than an STI.^25^ But in South Africa, visibility of cervical cancer is low, and—as in much of sub-Saharan Africa in general^30^—there is little knowledge about the impact of cervical cancer on female morbidity and mortality.^21^^,^^23^^,^^31^ The danger, then, was that parents would regard HPV vaccination as “non-essential,” leading to poor uptake.

Few national HPV vaccination programs have yet been initiated in southern Africa, largely because of the high cost of the vaccine. This is a particular concern for countries in the region that are ineligible for funding from Gavi, The Vaccine Alliance. South Africa has partially overcome these cost concerns thanks to political commitment to vaccination and the registration of a 2-dose—rather than a 3-dose—schedule. In early 2014, South Africa introduced a national program of HPV vaccination, with ambitious hopes of meeting high coverage targets.^7^

We undertook a process evaluation in April 2014 to assess the success of the first-dose campaign and identify practical challenges that could be addressed prior to implementation of the second-dose campaign. Key components of the vaccination campaign were evaluated and conclusions fed back to the implementing body—the National Department of Health (NDoH). While the aim of the evaluation was to identify and resolve problem areas in time for administration of the second dose, its findings also have broader relevance for strengthening the HPV vaccination program overall. In this article, our aim is to illustrate what implementation challenges were experienced introducing a new vaccine to a new target population, outside of the traditional clinic environment, and offer useful lessons for HPV vaccine programming not only in South Africa but also in similar settings elsewhere.

## METHODS

### Program Description

Initiated in 2014, South Africa's national HPV vaccination campaign is a public school-based initiative to provide free vaccination to all Grade 4 girls aged ≥9 years. In the 2014 vaccination campaign, a bivalent HPV vaccine was used, with a 2-dose schedule—the second dose is provided 6 months after the first. The campaign was housed within the relaunched Integrated School Health Program (ISHP)—a program jointly implemented by the NDoH and the Departments of Basic Education (DBE) and Social Development (DSD). Grade 4 was used to identify those most likely to be 9 years old, the youngest age cohort eligible for the vaccine. School attendance at primary school level is compulsory in South Africa and virtually universal.^32^

South Africa initiated a national public school-based initative to provide free HPV vaccination to all Grade 4 girls aged 9 years and over.

Prior to campaign initiation, a national task team headed by a dedicated national coordinator was formed to provide support to provincial, district, subdistrict, and school teams. All provinces prepared HPV implementation and vaccine distribution plans. The national Ministers of Health and Basic Education jointly convened meetings with school governing bodies, school principal organizations, and teacher unions at national level to explain the HPV vaccination campaign and secure agreement from them to proceed.

Social mobilization efforts involved the development of school-specific informed consent packages that included consent information, education, and communication (IEC) materials, such as posters, fact sheets, frequently asked questions, and a guide for educators (Figure). These packages were distributed by provincial DoH and DBE staff to the appropriate audiences (schools, parents, and government employees). In addition, informed consent forms were distributed in all 11 official languages of South Africa to some 18,000 public schools. Information about the campaign was placed on government websites and social media networks and relayed through broadcasts on national radio. The Health Minister's official launch of the campaign received wide television exposure on the national broadcaster's “Morning Live” breakfast show.

**FIGURE:**
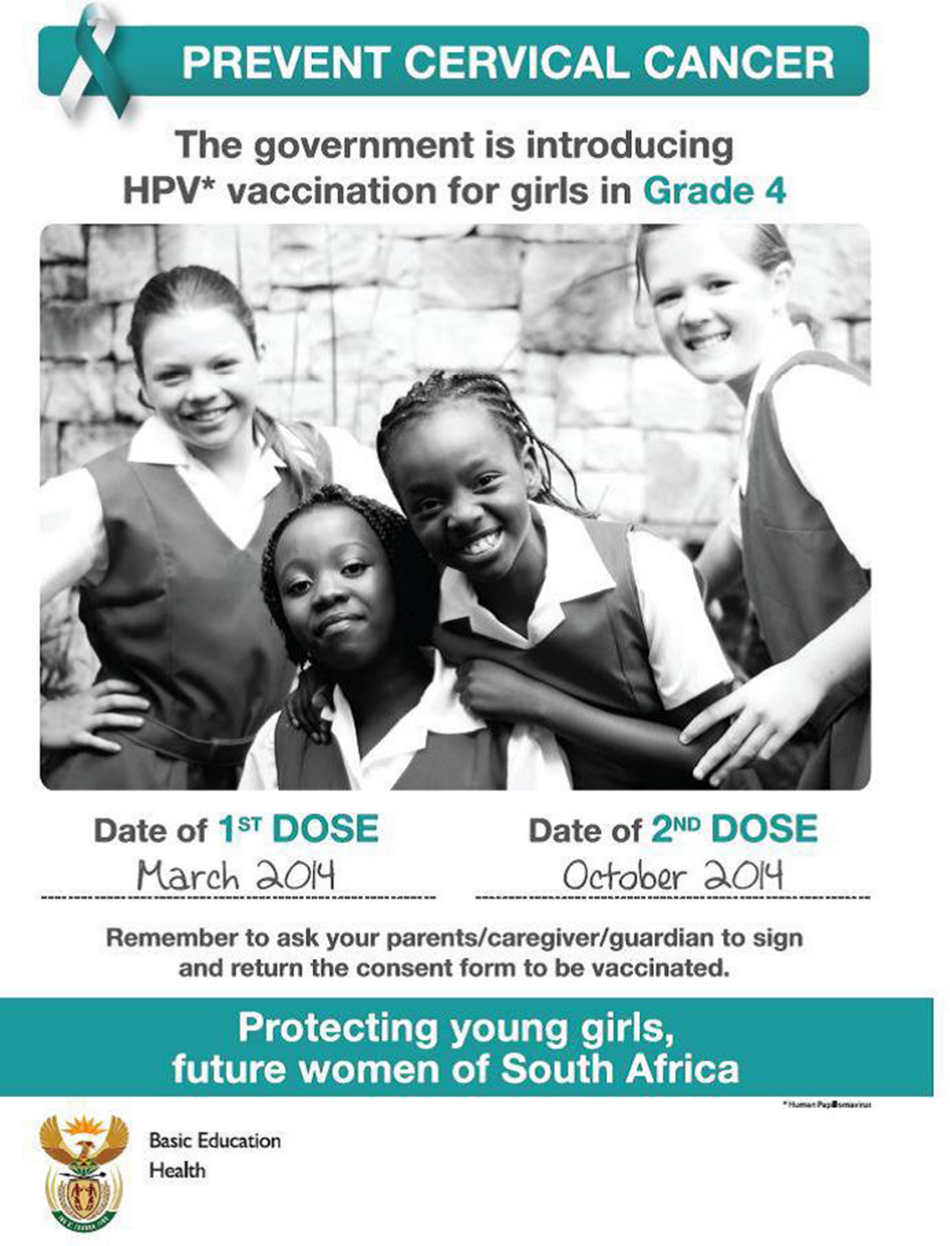
Social Mobilization Poster Distributed by the South African Department of Health During the 2014 HPV Vaccination Campaign Abbreviation: HPV, human papillomavirus

Training materials—developed by the NDoH with the support of partners—included a field guide and a set of training slides. A 2-day training session was held at national level and an additional 1-day training session was conducted for provincial, district, subdistrict, and facility-level teams.

On vaccination days, DoH vaccination teams visited assigned schools and implemented set procedures involving education, eligibility control, vaccination, data recording, and observation of vaccinated girls (physically separated from girls who were still awaiting vaccination). “Mop-up” visits were made where necessary to reach eligible girls who had been absent on the day of vaccination.

In terms of monitoring and evaluation, the NDoH developed a new school-based data subset linked to the District Health Information System (DHIS). Vaccination teams kept registers of vaccinated girls, completed weekly summary reports of all activities, and recorded adverse events in the DoH's routine adverse events (AE) reporting system. A target-driven strategy was adopted overall to encourage a strong focus on monitoring and reporting throughout the campaign.

### Study Design and Data Collection

We used a cross-sectional and mixed-methods approach, combining qualitative and quantitative data, to evaluate the first-dose HPV vaccination campaign. The 4 principal sources of information used in the evaluation are detailed below.

#### Review of Records and Materials

We reviewed all records and materials used in the planning and preparation of the campaign, as provided by the NDoH, at a 2-day post-campaign review and planning meeting. This group of primary sources included planning tools, training and social mobilization materials, core program materials (field guide, consent forms, invitation letters, data collection tools, and vaccination cards), summary reporting data (including adverse event reports), and presentations. Data were extracted from all reviewed sources in accordance with a standardized guide that had been developed beforehand.

#### Direct Observation of Vaccination Sessions

Three researchers observed a total of 7 vaccination events in 4 provinces—Gauteng, KwaZulu-Natal, Mpumalanga, and North West—in the final week of the first-dose campaign. Purposive sampling was used to ensure observation of vaccination activities in both rural and urban settings. To guide the observations, the researchers used a standardized NDoH tool designed to assess fidelity of implementation to the HPV Vaccination Campaign Field Guide. Observations specifically included assessments of microplans, social mobilization, vaccine session preparation and preparedness (including cold chain, safety considerations, and administrative procedures), vaccine administration (including eligibility determination and consent process, patient management, monitoring and flow, stock control, and vaccination team roles), and data and recordkeeping (Supplement 1).

#### Key Informant Interviews

Primary data were collected through key informant interviews with a total of 29 DoH officials involved in the campaign at provincial, district, and subdistrict levels. In each of the 9 provinces, 1 district was randomly selected by number, and within each district, the 2 subdistricts with the highest and lowest coverage were selected based on preliminary data. At provincial level, all 9 HPV vaccine campaign coordinators were interviewed, while at district level, only 8 coordinators were interviewed, as 1 district lacked an appointed coordinator. Owing to the lack of appointed coordinators in some subdistricts, only 12 coordinators at this level were interviewed. In addition, a total of 5 NDoH officials were purposively selected and interviewed.

Interviews (either telephonic or in person) were conducted by 1 researcher on the team. A semistructured interview guide was used, with open-ended questions focusing on factors influencing coverage, safety, adverse events following immunization (AEFI), data capture, and social mobilization (Supplement 2). All participants provided written informed consent for both the interview and the recording of interviews. The interviews lasted an average of 60 minutes, and were conducted in English and transcribed in full for analysis.

#### Assessment of Media Coverage

Media coverage of the HPV campaign was reviewed retrospectively for the period March 1 to April 30, 2014. This period began several days before the start of the campaign and ended several days after the campaign's conclusion. The review, conducted by an external company specializing in media analysis, included over 900 print, broadcast, and online media sources from 80 newspapers, 291 community publications, 95 magazines, 37 radio stations, and 13 television stations. The following search terms were used to identify relevant material: HPV vaccination, HPV schools vaccination, cervical cancer vaccine, HPV vaccine, and schools HPV.

### Evaluation Outcomes and Data Analysis

The main outcomes of interest in the evaluation were program coverage, vaccine safety, and factors that influence implementation of the program. The data used for the analysis were collected between March 10 and April 23, 2014, using NDoH HPV vaccination campaign data as of September 27, 2014. Program coverage was defined as: school coverage, age eligibility among Grade 4 girls (9 years or older on the date of the first-dose administration) at schools reached by vaccination teams, and age-eligible learner coverage—the term “learner” is used by the DBE and all other government departments to describe a student. The campaign had a target of 100% for school coverage, defined as the number of public schools—both ordinary primary, intermediary, and combined schools and “special schools,” which were equipped to educate learners who have special needs—with Grade 4 that were reached by the campaign as a percentage of the total number of public schools with Grade 4 in the country. The target for learner coverage was 80%, which program planners considered to be the threshold required for “herd immunity”^33^ although, increasingly, evidence supports even lower coverage as an adequate threshold.^34^ Learner coverage was defined as the number of Grade 4 girl learners 9 years and older who were vaccinated as a percentage of the total number of age-eligible learners (Grade 4 girl learners 9 years and older).

To assess safety, we reviewed all official AEFI reports from the campaign, alongside onsite vaccination observations (described earlier) of staff preparedness for AEFIs. In South Africa, the management of AEFIs for HPV is the same as for other vaccines, and includes 5 key steps: detection and reporting, investigation, collation and analysis of data, implementation of corrective measures, and evaluation of the surveillance and handling of the cases.^35^

We analyzed the factors influencing implementation of the program using a process evaluation framework with broad parameters,^36^ including planning and coordination; dose delivered by providers and dose received by target audience; recruitment through social mobilization, defined as a process of disseminating information and of gaining and sustaining involvement from all stakeholders; and media response. This allowed us to assess potential risks to sustained implementation of HPV vaccination programs of this scale in the future.

All program documentation provided by the NDoH was reviewed by 1 researcher who identified program strengths, weaknesses, and gaps, and assessed checklists—developed beforehand and completed during observation visits—for common themes. The same researcher reviewed and manually coded interview transcripts to identify common themes relating to challenges, risks, and successes in the implementation of the campaign. These themes were then sorted into a matrix in Microsoft Word and key findings summarized to capture the content of each theme.

The media analysis company hired to assess media coverage of the campaign objectively reviewed all identified media items for content, and categorized them according to the most likely overall perception of the reader. This categorization used a 3-point rating scale based on standardized measures of positive (favorable descriptions of the campaign), neutral (unbiased, mostly factual information about the campaign), or negative (negative language and examples used to describe the campaign). In the absence of data collected directly from parents and community members, the media analysis offered vital information on the shaping of public perceptions of the vaccine.

### Ethical Considerations

The study was reviewed and approved by the University of the Witwatersrand Human Research Ethics Committee. All participants provided written informed consent prior to participation.

## RESULTS

Since our evaluation of the first-dose phase of the 2014 campaign was designed mainly to extract lessons to improve implementation of the second-dose phase, the analysis of our findings focused on 2 main categories: (1) areas of success and (2) aspects in need of further strengthening. In presenting our findings below, we retain this conceptual division and consider in each category how the study outcomes were impacted.

### Campaign Successes

#### Planning and Coordination

The campaign was introduced in the context of a high-level political mandate, and interview data showed that the strong political commitment to the campaign was an important factor driving results. Campaign planning and coordination was managed centrally by a team of highly experienced, committed NDoH staff who established strong communication mechanisms at provincial and district levels to monitor progress and address challenges. All provinces and districts appointed coordinators who oversaw microplanning at the site level to project vaccine and resource needs. Coordination mechanisms were used to mobilize support from a range of partners, including nursing schools, the South African National Defence Force and developmental partners. Collectively, this commitment helped to counter some of the challenges posed by tight timeframes and limited budget resources for a campaign of this magnitude.

#### Coverage

Interviews, record reviews, and observations of vaccination events showed that subdistricts had developed a clear schedule to cover 100% of schools. Contrary to concerns that low knowledge and visibility of HPV and cervical cancer might affect uptake, overall coverage was high: 91% of schools (15,620 out of 17,175) were reached with vaccination sessions in total. This suggested that vaccination teams and planners had successfully overcome the logistical challenges that arose in reaching some schools, such as flooding and lack of transportation.

With regard to learner coverage, a total of 408,273 Grade 4 girls age-eligible for vaccination were reached—received informed consent packages—during the campaign, of whom 353,564 (86.6%) were vaccinated. The eligible girls who were not vaccinated (13.4%) included girls who had not received parental consent or were absent on the vaccination day or not medically eligible for the vaccine due to ill health on the day. In terms of the proportion of Grade 4 girls who were too young to meet the eligibility criteria, based on NDoH data available through August 25, 2014, about 12% of Grade 4 girls were age-ineligible to receive the vaccine during the March 2014 campaign (range by province: 5% to 17%).

The campaign reached about 408,000 age-eligible girls for vaccination, of whom about 87% were vaccinated.

#### Safety

Of the over 353,000 girls vaccinated in the campaign, only 10 case reports of AEFIs (0.003%) were received by the NDoH. All 10 of the cases were categorized as minor, time-limited events, such as a rash, abdominal pain, raised temperature, dizziness, nausea, and fainting. Five of the 10 cases began experiencing symptoms shortly after receiving vaccination while still under observation by vaccination staff. All 5 were accompanied to a health facility by a member of the vaccination team. Of the remaining 5 cases whose reactions began later in the day at home, 1 child was treated at home and the other 4 were taken to a health facility, where they were treated symptomatically for faintness, rash, or nausea and then observed and later discharged. Based on our analysis of the provincial post-campaign summary reports, we identified 2 additional, unreported cases of AEFIs. These learners experienced minor reactions—fainting and vomiting—and both were treated at the vaccination session by the same vaccinator.

Only 10 cases of adverse events were reported, all categorized as minor.

#### Dose Delivered and Received

Observations found that the management of individual vaccination sessions was generally well organized. Program organizers were able to tap into the knowledge of retired nurses, who had vast experience of participating in Expanded Programme on Immunisation (EPI) campaigns over the years, by including them in vaccination teams wherever possible. The sequence of required procedures flowed effectively—from education, to eligibility control, vaccination, data recording, and observation—and vaccination teams paid great attention to learner comfort and preparedness. Adequate supplies in the form of bundled single-dose vaccines were delivered to provinces in good time. Overall, vaccine supply was well managed, with cooler boxes provided for each vaccination team along with adequate non-gel ice packs to prevent freezing of the vaccines.

#### Media Response

In any vaccination endeavor, social mobilization has the potential to be supported by positive reporting or undermined by negative reporting in the mass media. Analysis of media coverage found that a total of 373 items on HPV vaccination were published or broadcast in the period March 1 to April 30, 2014, the majority (68%) online, with just under a third (28%) in print media and only 4% in broadcast media (radio and television). Over half (55%) of all media items were categorized as neutral, with 38% considered positive and only 7% designated as negative. Of the positive media items, most (70%) were released in March (the first month of the campaign), while 59% of negative coverage was released the following month, suggesting that after the initial time period, a shift in public discourse about the campaign may have occurred. We explore possible reasons for this shift below.

### Campaign Challenges

#### Planning and Coordination

Notwithstanding key successes, our assessment of the first-dose phase revealed some vulnerabilities in campaign planning. This planning process required the development of tools and materials at national level, and the coordination and training of hundreds of teams down to the subdistrict level, all of which was completed in an impressive 6-month period. Key informants identified training gaps in some districts and suboptimal use of NDoH microplanning tools (mainly due to lack of capacity in using Microsoft Excel worksheets). Fortunately, these vulnerabilities were offset by creative cross-program teamwork, which was evident from planning, to training, to vaccination implementation. Examples included teamwork in budget sharing and staff training, and the involvement of vaccine teams from a range of programs. For instance, in the Eastern Cape province, staff from EPI, ISHP, ward-based outreach teams, primary health care, and nongovernmental partners collaborated to form localized HPV vaccination teams.

Due largely to the ambitious planning and implementation time frame, the coordination of the range of key stakeholders was challenging. Delays in stakeholder engagement impacted social mobilization in some provinces. In particular, key informant interviews revealed that the DBE's participation in campaign planning was delayed; this limited the social mobilization that could be carried out in schools prior to the campaign. Observers noted that school readiness for the vaccination teams was also delayed in some cases. Despite a slow start, collaboration between NDoH and DBE improved at all levels over the course of the campaign, establishing a strong platform for future campaigns.

Delays in stakeholder engagement impacted social mobilization in some provinces.

Officially, the campaign was located within the ISHP, which has expertise in providing services in schools and coordinating with the DBE but lacks capacity in the crucial areas of cold chain management and campaign microplanning. Because it had only recently been relaunched in South Africa, staffing patterns in the ISHP still varied widely across the country, particularly at the provincial, district, and subdistrict levels. In districts where no ISHP staff were available for coordination roles, the role was filled by a mix of EPI program, primary health care, health promotion, and other specialist NDoH teams.

#### Coverage

Despite high levels of school coverage overall, we found a wide variation by subdistrict and isolated pockets of low coverage that key informants attributed to challenges experienced with informed consent and anti-vaccine activities (see below). In 2 subdistricts in KwaZulu-Natal and Mpumalanga, lows of 40% and 43% school coverage were reported, respectively. Unexpected changes made to the campaign start date also resulted in overlap with school holidays and examinations and impacted learner coverage in several districts.

Despite high levels of school vaccination coverage overall, we found wide variation by subdistrict and isolated pockets of low coverage.

The greatest challenge in assessing coverage was in the management and reporting of data that underpinned the program at the subdistrict and district levels. Despite efforts to assign schools to health district boundaries, rather than traditional educational districts, discrepancies emerged between school lists provided by the DBE and those formed by vaccination teams on the ground.

Data quality also emerged as a challenge, with data not properly cleaned and verified prior to reporting, largely owing to inadequate capacity and tight reporting timelines. However, in the Eastern Cape, the HPV coordinator maintained a parallel reporting system and was able to identify inconsistencies in the DHIS data compared with data maintained in the parallel system. The data registers and reporting forms used in the campaign may also have contributed to data inconsistencies. Although the vaccination register used to report each vaccination session included a more detailed age breakdown than required for a grade-targeted campaign (age <9 years and ≥9 is sufficient), it lacked a place to record totals that would account for all Grade 4 girls as either vaccinated or ineligible.

#### Safety

While the total number of AEFIs was encouragingly low, there were minor issues with how these were handled by campaign staff. Of the 10 reported AEFI cases, the majority of cases were taken by the vaccination team to a health facility for treatment rather than being treated at the vaccination site. While the reasons for this decision are not described in the documents reviewed, it raises the possibility that the vaccinators were not comfortable or confident enough to treat AEFIs onsite. Although health care providers are trained to manage AEFIs, they are seldom required to conduct emergency procedures.

The additional risk created by administering vaccines outside of a health facility was intended to be mediated by the training of vaccination teams, the provision of emergency trays, and back-up support from local emergency services. Our assessment found that the training materials designed to prepare providers for managing AEFIs were clear and comprehensive; however, in some areas, the period of training was too short, leaving providers ill-equipped to cope with an emergency situation. In addition, observers noted that in some schools the emergency trays were not uniformly complete—for example, they lacked syringes, sterile water, and other supplies and, in most cases, emergency services had not been informed of the location of vaccine campaigns, as recommended by the field guide.

#### Dose Delivered and Received

Data from NDoH records, observations, and interviews confirmed that substantial pre-campaign preparation went into reducing the risk of breaks in the cold chain, particularly of vaccines freezing. Despite these preparations, important deviations from optimal cold chain were noted on observation visits and in discussions with key informants. For example, observation visits found that cold chain technologies (freeze tags, fridge loggers) were not uniformly used as intended, and ice packs were not “conditioned” in all cases, resulting in the risk of vaccines freezing. In addition, power failures occurred in some settings, while in others, domestic refrigerators—which have a higher risk of freezing vaccines—were used to store vaccines instead of specialized vaccine refrigerators. Additionally, abbreviated training at the subdistrict level, particularly for pharmacists, may have impacted training on cold chain maintenance.

Monitoring data showed that, in most provinces, reported vaccine use exceeded the number of learners vaccinated. Countrywide, 369,542 single-dose vials were reported as used, whereas only 353,564 learners were reported as vaccinated—a difference of almost 16,000, suggesting high vaccine wastage. More than 4,500 vials were reported as damaged or missing, costing just under 3 million Rand (based on an estimate of 650 Rand per vial).

#### Recruitment and Media Response

Implementing a new vaccine among a new target population, especially when operating outside of the traditional EPI or pediatric environment, creates a number of unique challenges. The involvement of multiple stakeholders and the unpredictability of the wider social context complicate social mobilization. At the heart of the study recruitment process is the need to obtain informed consent for a child's vaccination from their parents or guardians, a logistical challenge in its own right. More than 17,000 school-specific informed consent packages were delivered by the NDoH to the provinces, and while these packages were successfully distributed overall, late delivery of packages in some provinces delayed vaccination start dates. Large numbers of children were involved in obtaining informed consent, which was sought by proxy. In this system, the child was tasked with acting as a “go-between” relaying information about the vaccination program to the parent, securing their signature on the informed consent form, and returning the form to school authorities in time for the scheduled vaccination day.

Because a measles vaccination campaign had been implemented in schools across the country just before the HPV campaign, parents were largely familiar with the consent process and, importantly, with the concept of vaccinating pre-adolescents. Nevertheless, according to key informants, this familiarity did not prevent misunderstandings and inconsistencies from arising. Additionally, the wording of the HPV campaign informed consent forms—“I hereby grant/do not grant permission for my child to receive 2 doses of the HPV vaccination”—confused many parents who believed the form referred to a social grant from the government. Parents were also reported to have been confused by the rollout of the *National Contraception and Fertility Planning Policy and Service Delivery Guidelines*^37^ and, in particular, the launch of contraceptive implants, which took place around the same time as the start of the HPV vaccination campaign. According to key informants, this confusion was responsible for some parents declining consent for vaccination. While potential confusion may have been countered by the social mobilization materials developed by the NDoH and by the television appearance of the Minister of Health at the launch of the HPV vaccine campaign, the extent and impact of the confusion is not known.

In terms of media coverage, only 27 items (7% of all media coverage) about the 2014 HPV campaign were found to be negative. A rough breakdown of coverage by media type enabled us to identify the sources of negative messaging about the campaign (Table)—just over half (51.9%) of the negative media appeared in print, compared with only 18.5% in broadcast media and 29.6% online (figures not shown).

**TABLE.** Breakdown of Media Coverage of the 2014 HPV Vaccination Campaign in South Africa by Media Type and Rating (N=373)

Media Type	Rating		
	Positive No. (%)	Neutral No. (%)	Negative No. (%)
Print (n=105)	34 (32.4)	57 (54.3)	14 (13.3)
Broadcast (n=16)	10 (62.5)	1 (6.2)	5 (31.3)
Online (n=252)	96 (38.1)	148 (58.7)	8 (3.2)
**Total**	**140 (37.5)**	**206 (55.2)**	**27 (7.3)**

Only 7% of all media coverage about the HPV vaccination campaign was found to be negative.

In terms of content, of the 27 negative items, a majority (63%) related to parental concerns over vaccine safety, while the remainder either highlighted the high cost of HPV vaccine in the private sector (22%) or were critical of the campaign's exclusion of boys (15%). Although social media— Facebook, email, and short messaging service [SMS] used on cell phones, among others—was not covered in the media assessment, anecdotal information suggests that anti-vaccine messaging disseminated through social media may have posed an important threat to the success of the campaign. One SMS communication circulating in Mpumalanga province during the campaign read:

*It's a matter of life and death. If you have a daughter or granddaughter of 9 years old please listen very carefully. The schools are giving out permission forms to have these 9 year old girls vaccinated against a virus called HPV. You should under NO circumstances do this! The vaccine is unstable. 32 women died in the U.S. from the vaccine. It's still in the experimental phase and not reliable. Please moms! If you love your daughters. Refuse! You may. The government cannot force you. Please warn everyone. PLEASE! Go have a look at the link and the other links on this Page* http://www.infowars.com/japan-withdraws-support-for-hpv-vaccines-due-to-infertility-side-effects. (translation from Afrikaans)

The web address in this message directs the reader to a U.S.-based website notorious for its publication of conspiracy theories, “fake news,”^38^ and anti-vaccination articles. While our evaluation of the 2014 HPV vaccination campaign was not designed to assess how public perceptions of the vaccine in South Africa were actually affected by media coverage, whether in mainstream or social media, it is noteworthy that in at least 1 province, key informants believed the negative coverage about vaccine safety had made some DoH staff nervous about vaccinating girls in this campaign. Additionally, in areas where misinformation and rumors were found to have been shared on social media, campaign staff reported a negative impact on parental consent.

## DISCUSSION

The first round of the South African national HPV campaign implemented in 2014 achieved high overall coverage, a good safety profile, and mostly positive implementation experience. Implementing a vaccine campaign of this size and complexity requires careful planning, adequate resources, a receptive target population, and effective coordination throughout. Our evaluation showed that campaign success depended on a wide range of expertise, particularly in the domains of school health services, cold chain management, vaccine microplanning, health promotion, social mobilization, and health informatics. Implementation was clearly facilitated by strong political leadership and intersectoral coordination. Where gaps did emerge, many had been anticipated prior to the campaign—such as human resource shortages and cold chain weaknesses. Assuming continued political commitment to HPV vaccination in South African schools, these logistical shortcomings could conceivably be addressed with relative ease in future campaigns.

Some concerns anticipated in early feasibility and acceptability research proved to be unfounded, while other concerns emerged during the campaign. While our evaluation did not assess the extent of community-level resistance to HPV vaccination based on fears of possible sexual disinhibition among vaccinated girls, even if some parents and community members had, in fact, given credit to this theory, it did not appear to gain any traction in this campaign. Similarly, virtually none of the negative media coverage identified in our evaluation focused on the sexual dimensions of HPV. Instead, the mainstream media attention was directed at vaccine safety, which dominated messaging on social media. Importantly, this messaging had a strong global imprint, as much of its content was sourced from anti-vaccination lobby groups and influential “victim” support groups based outside of South Africa.^39^^,^^40^ With an extensive online presence, these groups style themselves as global outposts of resistance to HPV vaccines.^41^ Their efforts to influence public discourse ignores or misinterprets extensive clinical safety data on the 2 commercially available HPV vaccines, Gardasil and Cervarix.^42^

In a study of a public-sector HPV vaccine introduction in Australia, social media was found to have had a substantial influence on acceptance of the vaccine program among parents and, subsequently, on uptake.^43^ As in many parts of the world, South African parents deciding about HPV vaccination are increasingly likely to search the Internet for information about the vaccine where they will encounter an overwhelming mix of fact, opinion, and misinformation offered by online web-based groups and social media.^44^ The challenge is that it is often difficult to assess the credibility of these sources. Furthermore, the user-generated content—a key feature of social media—encourages lay persons to engage with medical knowledge, selecting or rejecting information based on their personal “truths” or those of other online users.^45^ This type of content can effectively mobilize those who already have low levels of trust in conventional biomedicine. As the HPV vaccination program in South Africa matures, it will be important to monitor the influence of Internet-based anti-vaccination groups and social media conversations on local attitudes toward the vaccine.

Several implementation lessons emerged from the findings of this evaluation. First, a successful HPV vaccination campaign of this scale requires effective partnership building between government ministries, primarily those responsible for health, education, and social development. This is especially important in school settings receiving multiple health interventions. From a health service delivery perspective, the prospect of delivering HPV vaccination in South African schools as part of an integrated package of care for adolescents has been proposed. Such a package could include services as wide-ranging as screening for vision and hearing impairment, information on gender-based violence, and provision of condoms and tampons.^22^ This idea meshes well with the newly revitalized ISHP and with the new *DBE National Policy on HIV, STIs and TB*,^46^ which was released in June 2017. The latter proposes that a wide range of services be offered to learners at schools via mobile health units or alternative channels, including dual protection and other contraception, HIV counseling and testing, adolescent-friendly health services, and screening for STIs.^46^ In this context, vaccination teams would then need to manage competing programs and ensure that the roles of all stakeholders and partners are clarified and communicated from the outset. Particularly important is the need to formalize the crucial role of the DBE in providing strategic information to target schools and learners throughout the country; in communicating effectively with parents and the wider community, including school governing boards and teachers, about the campaign; and in supporting social mobilization efforts at all levels.

Successful school-based HPV vaccination campaigns require effective partnership building between government ministries.

Second, notwithstanding the high coverage attained in the first-dose phase of this campaign, reach and efficiency could have been further maximized with some simple adjustments to scheduling. For example, isolated, hard-to-reach schools could have been scheduled for vaccination in a different season when transport routes were not affected by rain. Furthermore, implementers could explore widening the interval between the first and second vaccine dose to 12 months, thereby necessitating only 1 campaign visit to schools each year. In the context of limited resources, consideration could also be given to eliminating or reducing mop-up visits. In the 2014 campaign, first-dose coverage exceeded the threshold needed for “herd immunity,” defined as vaccinating at least 80% of learners. Therefore, mop-up visits had little strategic value. In general, they should be undertaken only if a significant proportion of age-eligible girls are unvaccinated on the initial vaccination day. To save costs, unvaccinated girls could also be followed up through health centers instead of return visits to schools.^47^

Implementers could explore widening the interval between the first and second vaccine dose to 12 months so that schools will only need 1 campaign visit each year.

Third, the large discrepancy between reports of the number of vaccines used and the records of girls vaccinated signals a possible underestimate of vaccine coverage. However, this is difficult to confirm if a campaign has weaknesses in data quality, data reporting, and overall monitoring and evaluation planning and structure. Recommendations for addressing these weaknesses include taking steps to simplify vaccination registers and weekly summary sheets and considering electronic registers and a web-based platform for data reporting. Clear standard operating procedures for data verification should be developed to help clarify procedures at each step of data recording and define the roles for all staff involved in this process, from coordinators and team leaders to data capturers and information officers. If resources permit, experienced monitoring and evaluation staff could be hired for the 2-month duration of the campaign.

Fourth, gaps in training coverage should be closed and better training outcomes ensured to avoid the problems in data management and cold chain integrity that we observed. Since the 2014 first-dose campaign was implemented by highly skilled health care workers at all levels, formal training could be reduced to key, well-identified, critical issues for inclusion in any campaign-related training. In developing training modules and tools, particularly in the areas of cold chain management and vaccine handling, consideration should be given to replacing conventional, didactic approaches with more participatory, practical approaches. This change could help improve retention of information and build health provider confidence to manage cold chain requirements and deal with AEFIs, should they arise. Strategies like training health workers to train others—using a cascading training-of-trainers approach—could also help to improve training coverage overall and build in-country capacity using fewer resources, as found in a HPV vaccination demonstration project in Peru.^47^

Fifth, the existing informed consent process used in South African schools urgently requires rethinking. On several levels, reliance on an opt-in approach that depends on children giving the form to their parents and the parents returning the signed form to the school is problematic: misunderstandings are highly likely and unconsented vaccinations can occur in error. HPV vaccination programs in more than 30 low- and middle-income countries using opt-out consent models have reported achieving higher coverage.^48^ This approach is not always feasible, however, particularly in countries such as South Africa, where a historical legacy of unconsented medical interventions^49^ may generate suspicion of an opt-out model. However, wherever the legal framework allows, alternative strategies for seeking consent from parents should be explored. One possibility is the use of mHealth applications to distribute vaccination information to parents and check comprehension prior to securing consent.

Opt-out vaccine consent models have reported higher coverage than opt-in models.

The final lesson relates to the management of adverse events and the role of social mobilization more broadly. Overall, our evaluation findings on the 2 unreported AEFIs that occurred suggest that the 10 reported cases may have underestimated the total number of minor AEFIs from this campaign. Importantly, no severe AEFIs were reported, leading us to conclude that AEFIs appear to be a low probability occurrence in this setting. However, even a minor AEFI that is not adequately managed has the potential to deter concerned parents from consenting to vaccination, reduce second-dose uptake, or be influenced by anti-vaccination groups. The latter, particularly, can lead to the development of larger, more organized efforts to disseminate misinformation and undermine public trust in the HPV vaccine. Uptake of the HPV vaccine could be significantly impacted by such a risk, unless it is countered by a sustained, proactive approach to tackle misinformation in a variety of online spaces, including social media. Indeed, a media rapid response plan, prepared prior to implementation, should ideally form an integral part of the management of AEFIs. This plan should include public health messaging that conveys complex vaccination information in simple, accessible language, and be flexible enough to be activated at any level to respond in real-time to negative media coverage.^11^

Beyond such targeted responses to an emergency, effective social mobilization should be approached as a long-term investment for building community support. Managing community perceptions of safety is a crucial issue for all vaccine programs, particularly with the introduction of new vaccines that are not well known among the target population and broader community. In a survey of HPV program managers in 19 low- and middle-income countries, the most frequently reported obstacles to HPV vaccination were “erroneous perceptions of population related to the vaccine's safety and efficacy.”^12^ Given the profound changes in the media landscape in the past decade alone, it is time for program designers to explore innovative methods not normally used in public health communication. Possibilities include using the personal narrative format that anti-vaccination groups have appropriated so successfully, and greater use of digital applications that encourage users to interact directly with material, by sharing, commenting on, or uploading content.^50^

### Limitations

Our evaluation had some limitations. The generalizability of our conclusions may be somewhat restricted by the limited sampling of key informants and the purposeful sampling used to select sites for the direct observation of vaccination sessions. In addition, only 1 researcher was responsible for conducting the document review; ideally a second researcher would have reviewed the same set of documents, thereby allowing for comparison and confirmation of results and strengthening the reliability of conclusions.

## CONCLUSION

Evaluation of the 2014 campaign showed that implementation of a national school-based HPV vaccination campaign at scale was successful in this setting. Additional improvements to the storage and monitoring of vaccine doses and the informed consent processes, along with clearer stakeholder roles, will support optimization of school-based vaccination campaigns. While the impact of a national HPV vaccine campaign on cervical cancer will only be seen in the decades to come, as these early cohorts of vaccinated girls reach adulthood, the benefits of reducing HPV infections at a population level will be evident much sooner, for example, in declines in the prevalence of genital warts.^51^ The eventual integration of school-based HPV vaccination into routine EPI programming is a long-term project, and implementers need to be able to deliver a logistically complex intervention across multiple settings to reach high coverage every year. Evaluations contribute valuable lessons that help programs build capacity, decrease the burden on staff, reduce costs, and improve overall efficiency, so that the broader preventative potential of the HPV vaccine may be fully realized.

## Supplementary Material

18-00090-Scorgie-Supplement2.doc

18-00090-Scorgie-Supplement1.doc

## Notes

**Peer Reviewed**

**Competing Interests:** Sinead Delany-Moretlwe, Karen Kelley, Shamagonam James, Fiona Scorgie, Nicolette Naidoo, Admire Chikandiwa, and Helen Rees report grants from the U.S. President's Emergency Plan for AIDS Relief (PEPFAR) through the United States Agency for International Development (USAID), during the study period; Hasina Subedar, Nonhlanhla Dlamini, and Yogan Pillay are employed by the South African National Department of Health, which implemented the project evaluated by the study.

**First Published Online:** August 24, 2018

## References

[1] Ferlay J Soerjomataram I Dikshit R. Cancer incidence and mortality worldwide: sources, methods and major patterns in GLOBOCAN 2012. Int J Cancer. 2015;136(5):E359–E386. 10.1002/ijc.29210. 25220842

[2] van Schalkwyk SL Maree JE Dreyer Wright SC. Cervical cancer: the route from signs and symptoms to treatment in South Africa. Reprod Health Matters. 2008;16(32):9–17. 10.1016/S0968-8080(08)32399-4. 19027618

[3] Botha MH Dochez C. Introducing human papillomavirus vaccines into the health system in South Africa. Vaccine. 2012;30(suppl 3):C28–C34. 10.1016/j.vaccine.2012.03.032. 22939017

[4] Williamson AL Passmore J-A Rybicki E Marais D. Human papillomavirus (HPV) infection in Southern Africa: prevalence, immunity, and vaccine prospects. IUBMB Life. 2002;53(4-5):253–258. 10.1080/15216540212654. 12121005

[5] Kelly HA Ngou J Chikandiwa A; HARP Study Group. Associations of Human Papillomavirus (HPV) genotypes with high-grade cervical neoplasia (CIN2+) in a cohort of women living with HIV in Burkina Faso and South Africa. PLoS One. 2017;12(3):e0174117. 10.1371/journal.pone.0174117. 28333966 PMC5363860

[6] Denny LA Franceschi S de Sanjosé S. Human papillomavirus, human immunodeficiency virus and immunosuppression. Vaccine. 2012;30(suppl 5):F168–174. 10.1016/j.vaccine.2012.06.045 23199960

[7] Botha MH Richter KL. Cervical cancer prevention in South Africa: HPV vaccination and screening both essential to achieve and maintain a reduction in incidence. S Afr Med J. 2014;105(1):33–34. 10.7196/SAMJ.9233. 26046160

[8] Batra P Kuhn L Denny L. Utilisation and outcomes of cervical cancer prevention services among HIV-infected women in Cape Town. S Afr Med J. 2010 100(1):39–44. 20429487

[9] Botha MH Dreyer G. Guidelines for cervical cancer screening in South Africa. Southern African Journal of Gynaecological Oncology. 2017;9(1):8–12. http://www.sajgo.co.za/index.php/sajgo/article/view/253

[10] World Health Organization (WHO). Human papillomavirus vaccines WHO position paper. Wkly Epidemiol Rec. 2009;84(15):118–131. http://www.who.int/wer/2009/wer8415.pdf. 19360985

[11] World Health Organization (WHO). HPV Vaccine Communication. Special Considerations for a Unique Vaccine: 2016 update. Geneva: WHO; 2017. http://apps.who.int/iris/bitstream/handle/10665/250279/WHO-IVB-16.02-eng.pdf?sequence=1. Accessed June 15, 2018.

[12] Ladner J Besson MH Audureau E Rodrigues M Saba J. Experiences and lessons learned from 29 HPV vaccination programs implemented in 19 low and middle-income countries, 2009–2014. BMC Health Serv Res. 2016;16(1):575. 10.1186/s12913-016-1824-5. 27737666 PMC5062879

[13] Gallagher KE Howard N Kabakama S. Lessons learnt from human papillomavirus (HPV) vaccination in 45 low- and middle-income countries. PLoS One. 2017;12(6):e0177773. 10.1371/journal.pone.0177773. 28575074 PMC5456063

[14] Botha MH van der Merwe FH Snyman LC Dreyer G. The vaccine and cervical cancer screen (VACCS) project: acceptance of human papillomavirus vaccination in a school-based programme in two provinces of South Africa. S Afr Med J. 2014;105(1):40–43. 10.7196/SAMJ.8419. 26046162

[15] Moodley I Mubaiwa V Tathiah N Denny L. High uptake of Gardasil vaccine among 9–12-year-old schoolgirls participating in an HPV vaccination demonstration project in KwaZulu-Natal Province. S Afr Med J. 2013;103(5):318–321. 10.7196/SAMJ.6414. 23971122

[16] Msyamboza KP Mwagomba BM Valle M Chiumia H Phiri T. Implementation of a human papillomavirus vaccination demonstration project in Malawi: successes and challenges. BMC Public Health. 2017;17(1):599. 10.1186/s12889-017-4526-y. 28651574 PMC5485697

[17] Brotherton JML Fridman M May CL Chappell G Saville AM Gertig DM. Early effect of the HPV vaccination programme on cervical abnormalities in Victoria, Australia: an ecological study. Lancet. 2011;377(9783):2085–2092. 10.1016/S0140-6736(11)60551-5. 21684381

[18] Dorji T Tshomo U Phuntsho S. Introduction of a national HPV vaccination program into Bhutan. Vaccine. 2015;33(31):3726–3730. 10.1016/j.vaccine.2015.05.078. 26057136

[19] LaMontagne DS Barge S Le NT. Human papillomavirus vaccine delivery strategies that achieved high coverage in low- and middle-income countries. Bull World Health Organ. 2011;89(11):821–830B. 10.2471/BLT.11.089862. 22084528 PMC3209730

[20] Markowitz LE Hariri S Lin C. Reduction in human papillomavirus (HPV) prevalence among young women following HPV vaccine introduction in the United States, National Health and Nutrition Examination Surveys, 2003–2010. J Infect Dis. 2013;208(3):385–393. 10.1093/infdis/jit192. 23785124

[21] Delany-Moretlwe S Venables E Rees H MacPhail C. Introducing the HPV vaccine into South Africa: acceptability and feasibility. Paper presented at: 27th International Papillomavirus Conference and Clinical Workshop; September 17–20, 2011; Berlin, Germany.

[22] MacPhail C Venables E Rees H Delany-Moretlwe S. Using HPV vaccination for promotion of an adolescent package of care: opportunity and perspectives. BMC Public Health. 2013;13(1):493. 10.1186/1471-2458-13-493. 23692596 PMC3681713

[23] Katz IT Nkala B Dietrich J. A qualitative analysis of factors influencing HPV vaccine uptake in Soweto, South Africa among adolescents and their caregivers. PLoS One. 2013;8(8):e72094. 10.1371/journal.pone.0072094. 24023613 PMC3758285

[24] Francis SA Battle-Fisher M Liverpool J. A qualitative analysis of South African women's knowledge, attitudes, and beliefs about HPV and cervical cancer prevention, vaccine awareness and acceptance, and maternal-child communication about sexual health. Vaccine. 2011;29(47):8760–8765. 10.1016/j.vaccine.2011.07.116. 21855591

[25] Harries J Moodley J Barone MA Mall S Sinanovic E. Preparing for HPV vaccination in South Africa: key challenges and opinions. Vaccine. 2009;27(1):38–44. 10.1016/j.vaccine.2008.10.033. 18977271

[26] Mamo L Epstein S. The pharmaceuticalization of sexual risk: vaccine development and the new politics of cancer prevention. Soc Sci Med. 2014;101:155–165. 10.1016/j.socscimed.2013.11.028. 24560236

[27] Larson HJ Jarrett C Eckersberger E Smith DMD Paterson P. Understanding vaccine hesitancy around vaccines and vaccination from a global perspective: a systematic review of published literature, 2007–2012. Vaccine. 2014;32(19):2150–2159. 10.1016/j.vaccine.2014.01.081. 24598724

[28] Casper MJ Carpenter LM. Sex, drugs, and politics: the HPV vaccine for cervical cancer. Sociol Health Illn. 2008;30(6):886–899. 10.1111/j.1467-9566.2008.01100.x. 18761509

[29] de Bruin WE Panday-Soobrayan S. Learners' perspectives on the provision of condoms in South African public schools. AIDS Care. 2017;29(12):1529–1532. 10.1080/09540121.2017.1327647. 28509570

[30] Cunningham MS Davison C Aronson KJ. HPV vaccine acceptability in Africa: a systematic review. Prev Med. 2014;69:274–279. 10.1016/j.ypmed.2014.08.035. 25451327

[31] Mokhele I Evans D Schnippel K Swarts A Smith JS Firnhaber C. Awareness, perceived risk and practices related to cervical cancer and Pap smear screening: a cross-sectional study among HIV-positive women attending an urban HIV clinic in Johannesburg, South Africa. S Afr Med J. 2016;106(12):1247–1253. 10.7196/SAMJ.2017.v106i12.11224. 27917772

[32] Statistics South Africa. General Household Survey, 2015. Statistical Release P0318. Pretoria, South Africa: Statistics South Africa; 2016. https://www.statssa.gov.za/publications/P0318/P03182015.pdf. Accessed June 15, 2018.

[33] Fine P Eames K Heymann DL. “Herd immunity”: a rough guide. Clin Infect Dis. 2011;52(7):911–916. 10.1093/cid/cir007. 21427399

[34] Drolet M Bénard É Boily MC. Population-level impact and herd effects following human papillomavirus vaccination programmes: a systematic review and meta-analysis. Lancet Infect Dis. 2015;15(5):565–580. 10.1016/S1473-3099(14)71073-4. 25744474 PMC5144106

[35] The Expanded Programme on Immunisation in South Africa. National Department of Health South Africa. *Vaccinator's Manual: “Immunisation that Works.” Expanded Programme on Immunisation in South Africa (EPI-SA)*. Love Them, Protect Them, Immunise Them. 4th ed. Pretoria, South Africa: National Department of Health South Africa: 2012.

[36] Steckler A Linnan L, eds. Process Evaluation for Public Health Interventions and Research. San Francisco: Jossey-Bass; 2002.

[37] Department of Health, Republic of South Africa. National Contraception and Fertility Planning Policy and Service Delivery Guidelines. Pretoria, South Africa: Department of Health; 2012. https://www.health-e.org.za/wp-content/uploads/2014/05/ContraceptionPolicyServiceDelGuidelines2013.pdf. Accessed August 4, 2018.

[38] Gonzalez A Schulz D. Helping truth with its boots: accreditation as an antidote to fake news. Yale Law Journal Forum. 2017; 127:315–366. http://www.yalelawjournal.org/forum/helping-truth-with-its-boots. Published October 9, 2017. Accessed June 15, 2018.

[39] Wilson R Paterson P Larson HJ. The HPV Vaccination in Japan: Issues and Options. A Report of the CSIS Health Policy Center. Washington, DC: Center for Strategic and International Studies; 2014. https://www.csis.org/analysis/hpv-vaccination-japan. Accessed June 15, 2018.

[40] Larson HJ Wilson R Hanley S Parys A Paterson P. Tracking the global spread of vaccine sentiments: the global response to Japan's suspension of its HPV vaccine recommendation. Hum Vaccin Immunother. 2014;10(9):2543–2550. 10.4161/21645515.2014.969618. 25483472 PMC4977439

[41] Bean SJ. Emerging and continuing trends in vaccine opposition website content. Vaccine. 2011;29(10):1874–1880. 10.1016/j.vaccine.2011.01.003. 21238571

[42] Richter KL Dreyer G Lindeque BG Botha MH; South African HPV Advisory Board. HPV vaccine: can we afford to hesitate? S Afr Med J. 2014;104(8):522–523. 10.7196/SAMJ.8449. 26307795

[43] Watson M Shaw D Molchanoff L McInnes C. Challenges, lessons learned and results following the implementation of a human papilloma virus school vaccination program in South Australia. Aust N Z J Public Health. 2009;33(4):365–370. 10.1111/j.1753-6405.2009.00409.x. 19689598

[44] Burnett RJ Larson HJ Moloi MH. Addressing public questioning and concerns about vaccination in South Africa: a guide for healthcare workers. Vaccine. 2012;30(suppl 3):C72–C78. 10.1016/j.vaccine.2012.03.037. 22939026

[45] Kata A. Anti-vaccine activists, Web 2.0, and the postmodern paradigm – an overview of tactics and tropes used online by the anti-vaccination movement. Vaccine. 2012;30(25):3778–3789. 10.1016/j.vaccine.2011.11.112. 22172504

[46] Department of Basic Education (DBE). DBE National Policy on HIV, STIs and TB. Pretoria, South Africa; 2017. https://www.gov.za/sites/www.gov.za/files/41024_gon777.pdf. Accessed July 10, 2018.

[47] PATH, Instituto de Investigación Nutricional, Ministerio de Salud de Peru, Estrategia Sanitaria Nacional de Inmunizaciones. HPV Vaccination in Latin America: Lessons Learned From a Pilot Program in Peru. Seattle: PATH; 2010.

[48] Kabakama S Gallagher KE Howard N. Social mobilisation, consent and acceptability: a review of human papillomavirus vaccination procedures in low and middle-income countries. BMC Public Health. 2016;16:834. 10.1186/s12889-016-3517-8. 27543037 PMC4992325

[49] Open Society Foundation. Against her will: forced and coerced sterilization of women worldwide. https://www.opensocietyfoundations.org/publications/against-her-will-forced-and-coerced-sterilization-women-worldwide. Published October 4, 2011. Accessed 7 Dec 2017.

[50] Betsch C Brewer NT Brocard P. Opportunities and challenges of Web 2.0 for vaccination decisions. Vaccine. 2012;30(25):3727–3733. 10.1016/j.vaccine.2012.02.025. 22365840

[51] Ali H Donovan B Wand H. Genital warts in young Australians five years into national human papillomavirus vaccination programme: national surveillance data. BMJ. 2013;346:f2032. 10.1136/bmj.f2032. 23599298

