# Supplementary material for: Human Papillomavirus Vaccine Introduction in South Africa: Implementation Lessons From an Evaluation of the National School-Based Vaccination Campaign
Source: Glob Health Sci Pract. 2018 Oct 3;6(3):425–38. doi: 10.9745/GHSP-D-18-00090 (PMC6172125; doi:10.9745/GHSP-D-18-00090)
Supplement: 18-00090-Scorgie-Supplement2.doc [file 18-00090-Scorgie-Supplement2.doc]

| *Supplement 2. Interview Guide for Key Informants*  ***Mid-Stream Assessment of South Africa’s HPV Vaccination Campaign*** |
| --- |

***NOTE: This is a draft document which may be refined on completion of record reviews.***

***Instructions: Please fill in this KII INTERVIEW GUIDE, NEATLY, using a BLACK PEN. Any corrections or alterations must be signed and dated as per GCP. If you take notes in a separate notebook, be sure to attach these to these pages.***

| AUDIO FILE NAME |  |
| --- | --- |

Instructions: Please fill information in the table below before an interview

| **ITEM** | **OPTION** | **ANSWER (provide count)** |
| --- | --- | --- |
| **SEX** | MALE |  |
|  | FEMALE |  |
| **Respondent category** | National DOH |  |
|  | Provincial DOH |  |
|  | District DOH |  |
|  | Educator |  |
|  | Parent/community member |  |
|  | Other stakeholder, specify |  |
| **Province** | EC |  |
|  | FS |  |
|  | GP |  |
|  | KZN |  |
|  | Limpopo |  |
|  | MP |  |
|  | NC |  |
|  | NW |  |
|  | WC |  |

**Interviewer preparation checklist (answer YES or NO or N/A)**

| Is the interview room private and quiet? *(Put the “please observe silence; interview on progress” sign on the door interview room and walls on corridor)* Yes / No |  |
| --- | --- |
| Is the audio recorder working? Yes / No |  |
| Do you have the correct version of the interview script? Yes / No |  |

**Ethics checklist (Answer Yes/No)**

| Have you explained the purpose of the research? |  |
| --- | --- |
| Have you explained issues related to confidentiality? |  |
| Have you explained how long the interview will take? |  |
| Have you tried to moderate expectations (i.e. no promise of payment, reward or immediate benefit?) |  |
| Have you obtained informed consent form? |  |
| Have you explained that the language of interview is English? |  |

Do you have any questions before we start?

SUMMARISE QUESTIONS IN BOX BELOW

|  |
| --- |

**Can we record this interview □ Yes □No**

If no, do not record but take detailed notes

**Introduction:**

Thank you for volunteering to discuss the implementation of the 1^st^ dose of the HPV Vaccination Campaign. The purpose of our discussion is to identify factors that facilitated or hindered success in the campaign.

The discussion today will contribute to the government’s efforts to improve delivery of the 2^nd^ vaccine dose in October 2014.

| **THEME** | **Broad research question *(Do NOT Read this out; please fill in the answer based on the response given. If in doubt consult supervisor)*** | **Probes *(use these suggested probes to stimulate conversation and direct the responses – do not rely solely on these questions, but continue to probe each response, asking for examples and details using the terms: WHAT, WHERE, WHEN, WHY, HOW)*** |
| --- | --- | --- |
| **Perceptions of success** | **Do you think that the HPV vaccination campaign has been successful?** | In what way?  What in your experience were successes? What are areas for strengthening?  Explore what are seen as measures of success by respondents – do they spontaneously mention coverage and safety? |
| **Assessment of planning and preparation for the campaign** | **Do you think that sufficient attention was given (in this district) to planning, preparing and promoting of the campaign?**  **What were areas of success?**  **What were areas that require further attention?**  **Are there any things that you would recommend be done differently in the next round?**  **Could issues related to planning, preparing and promoting the campaign have influenced success/coverage/safety?** | Depending on type of respondent, for DoH officials probe the following: Preparation and use of   1. HPV planning tools 2. Provincial HPV micro plans 3. Resource allocation 4. Education & Training of vaccinators & educators 5. Vaccine supply 6. Cold chain issues   All respondents, probe for comments on   1. Social mobilisation plans and activities 2. Availability of communication materials 3. Involvement, coordination and support and role of strategic partners |
| **Assessment of delivery of vaccination in schools** | **Do you think that the vaccination days themselves were successful? In what way?**  **Were there any challenges with scheduling of visits and/or access to schools? How was this addressed?**  **Were there any issues associated with determining eligibility and/or the consent process? How were these addressed?**  **Was sufficient education provided about the vaccine, vaccine safety and need for 2^nd^ dose?**  **Were there any issues associated with giving the vaccine itself? How were these addressed?**  **Were there any safety concerns or adverse events?**  **What recommendations do you have for implementation of the 2^nd^ dose?** | Probe for the following   - Role of the vaccinators, teachers, other school officials and School Governing Boards - Determining eligibility (age, grade, pregnancy status, allergies) - Consent process – how was it implemented, were there barriers to form completion, what were these etc - Education of girls and their parents about HPV vaccine and cervical cancer - Counselling girls to complete the 2 doses - Educate community about cervical cancer prevention - Adverse event management and reporting |
| **Assessment of monitoring, evaluation and coordination** | **From your perspective, was the programme well implemented and coordinated?**  **Did all stake-holders receive sufficient communication?**  **For programme implementers only:**  **Were monitoring and evaluation tools completed as planned?**  **Were they easy to complete?**  **Did they assist with guiding the programme**  **Are there any additional tools that are needed?**  **Are there any tools that you would drop?**  **Other comments** | Probe for *challenges, factors that facilitated success, and opportunities for improvement in each of the following areas:*   - Sub-district level - District Level - Province level - National level |

Thank you very much for your time. Do you have any additional comments or questions for me?

|  |
| --- |
