# Supplementary material for: Human Papillomavirus Vaccine Introduction in South Africa: Implementation Lessons From an Evaluation of the National School-Based Vaccination Campaign
Source: Glob Health Sci Pract. 2018 Oct 3;6(3):425–38. doi: 10.9745/GHSP-D-18-00090 (PMC6172125; doi:10.9745/GHSP-D-18-00090)
Supplement: 18-00090-Scorgie-Supplement1.doc [file 18-00090-Scorgie-Supplement1.doc]

| *Supplement 1. Observation Checklist**Mid-Stream Assessment of South Africa’s HPV Vaccination Campaign* |
| --- |

# HPV Vaccination Intra-campaign Monitoring Tool

| **DATE ____ / ____ / ____ 2014** | **PROVINCE:** | **District Visited:** |
| --- | --- | --- |
|  |  |  |
| **Names of Persons Interviewed during visit:** | **Designation** | **Subdistrict Visited:** |
|  |  |  |
|  |  | **Collection sites visited:** |
|  |  | **1.** |
|  |  | **2.** |
|  |  | **Schools visited:** |
|  |  | **1.** |
|  |  | **2.** |

|  |  | **Observed / verified (Yes/No)** | **Comments** |  |
| --- | --- | --- | --- | --- |
|  | **Micro plans** : Review District micro plans |  |  |  |
| 1.1 | Does the province have district, sub-district and collection site micro plans? | (Yes/No) |  |  |
| 1.2 | Are the micro plans complete (do they cover all the eligible schools in the district – check number of schools on EMIS)? | (Yes/No) |  |  |
| 1.3 | Is progress with the implementation of district, sub-district and collection site micro plans monitored? | (Yes/No) |  |  |
| 1.4 | Are corrective measures applied where targets are not met? | (Yes/No) |  |  |
| 1.5 | Is the district meeting the set targets of schools to be visited? | (Yes/No) |  |  |
| 1.6 | Are the target number of girls to be vaccinated reached? | (Yes/No) |  |  |
| 1.7 | If no record reasons: | (Yes/No) |  |  |
| 1.8 | Are there plans to achieve targets not attained for both schools and girls: | (Yes/No) |  |  |
|  | **SOCIAL MOBILISATION** |  |  |  |
| 2.1 | Were meetings held with: | (Yes/No) | Who convened the meeting and what was the composition of attendees? |  |
|  | 1. School Principals & Teachers | (Yes/No) |  |  |
|  | 1. School governing bodies | (Yes/No) |  |  |
|  | 1. Parent Teacher Associations | (Yes/No) |  |  |
|  | 1. Communities | (Yes/No) |  |  |
|  | 1. Traditional leaders | (Yes/No) |  |  |
|  | 1. NGOs & CBOs |  |  |  |
| 2.2 | Was there joint planning between Health and DB E regarding campaign (vaccine, dates, access to schools and learners), | (Yes/No) |  |  |
| 2.3 | Were IEC material (flyers, pamphlets, and posters) available?  How were these used? | (Yes/No) |  |  |
| 2.3 | Were the IEC materials distributed with the Consent forms and Vaccination cards.? | (Yes/No) |  |  |
| 2.4 | Were Consent forms available? | (Yes/No) |  |  |
| 2.5 | Were Vaccination Registers received? | (Yes/No) |  |  |
| 2.6 | Were HPV Vaccination Cards received? | (Yes/No) |  |  |
| 2.7 | Were HPV Consent Forms distributed to the schools before the HPV Campaign? | Yes/No) | . |  |
| **3** | **PREPARATION FOR VACCINATION SESSION** |  |  |  |
| 3.1 | Is there a Field Guide/flipcharts for HPV teams? | (Yes/No) |  |  |
| 3.2 | Are there sufficient HPV vaccines for the team? | (Yes/No) |  |  |
| 3.3 | Are there adequate quantities of syringes and needles? | (Yes/No) |  |  |
| 3.4 | Are there enough cooler boxes for this team (2 per team)? | (Yes/No) |  |  |
| 3.5 | Are there enough ice packs? (at least 2 sets)  Water based not gel packs. | (Yes/No) |  |  |
| 3.6 | How much time was taken to prepare and pack the cooler boxes? | (Yes/No) | Start time  Finish time  Total time |  |
| 3.7 | How much time did it take to get to the first vaccination point (one-way) | (Yes/No) | Start time  Finish time  Total time |  |
| 3.8 | Are there Vaccination Registers; adequate Vaccination Cards and, and weekly summary sheets? | (Yes/No) |  |  |
| 4 | **PREPAREDNESS AND SET UP AT SCHOOL** | ( |  |  |
| 4.2 | Is there a dial thermometer for main the cooler box that has most vaccines? | (Yes/No) |  |  |
| 4.3 | Is the temperature in the cooler box between 2-8^o^C | (Yes/No) |  |  |
| 4.4 | Are the ice packs conditioned (rattling, not frozen)? | (Yes/No) |  |  |
| 4.5 | Are there enough Sharps disposal containers? | (Yes/No) |  |  |
| 4.6 | Are there waste disposal bags/box for other waste e.g. cotton balls | (Yes/No) |  |  |
| 4.7 | Is there a hand disinfectant spray? | (Yes/No) |  |  |
| 4.8 | Is there a place for the girls to sit while being injected with the vaccine? | (Yes/No) |  |  |
| 4.9 | Is there an emergency tray | (Yes/No) |  |  |
| 4.10 | Is the procedure for reporting AEFI understood?  (Ask health care worker to explain - procedure) | (Yes/No) |  |  |
| 4.11 | Are the AEFI reporting forms available? | (Yes/No) |  |  |
| 5 | **VACCINATION SESSION** | (Yes/No) |  |  |
| 5.1 | Is there vaccination station set up and organised? | (Yes/No) |  |  |
| 5.2 | Is the post well organised with good client flow? | (Yes/No) |  |  |
| 5.3 | Are there sufficient staff on the team? | (Yes/No) |  |  |
| 5.4 | Is there a designated area to monitor the girls post vaccination? | (Yes/No) |  |  |
| 5.5 | Are the girls afraid of vaccination well managed? Is there an effort to put aside girls who are afraid to be vaccinated aside and reassure them? | (Yes/No) |  |  |
| 5.6 | Are parents accompanying their girls for vaccination well accommodated? | (Yes/No) |  |  |
| **6** | **BRIEFING SESSION**  ***(Please ask question 5.1 to 5.4 to 5 girls )*** |  |  |  |
| 6.1 | Was there a briefing session before vaccination? | (Yes/No) |  | |
| 6.2 | Are girls welcomed and treated respectfully | (Yes/No) |  | |
| 6.3 | Was the inclusion/exclusion criteria explained e.g. severe febrile illness and girls younger than 9 years. | (Yes/No) |  | |
| 6.4 | HPV vaccine, no. Of doses, side effects explained and girls asked any questions | (Yes/No) |  | |
| **7** | **VACCINE ADMINISTRATION** |  |  | |
| 7.1 | Is the parent consent confirmed before vaccinating the girls? | (Yes/No) |  | |
| 7.2 | Are VVMs checked before a vaccine is used? | (Yes/No) |  | |
| 7.3 | Was temperature of cooler box between 2-8^O^C? (Check the thermometer) | (Yes/No) |  | |
| 7.4 | Are there syringes pre-filled with the vaccine? | (Yes/No) |  | |
| 7.5 | Are the girls asked to keep arm relaxed (arm exercises) | (Yes/No) |  | |
| 7.6 | Is the vaccine administered intramuscularly in deltoid muscle of left upper arm. ( Right if left handed) | (Yes/No) |  | |
| 7.7 | Is the needle (uncapped) and syringe disposed of in sharps container? | (Yes/No) |  | |
| 7.8 | Are doses recorded correctly on the Register? | (Yes/No) |  | |
| 7.9 | Is the HPV Vaccination card filled in correctly? | (Yes/No) |  | |
| 7.10 | Is the date for the next dose recorded in the register and the vaccination card | (Yes/No) |  | |
| 7.11 | Are all the fields in the register and weekly summary sheet filled in correctly | (Yes/No) |  | |
| 7.12 | Are the girls given date of next dose | (Yes/No) |  | |
| 7.13 | Are the weekly summary sheets forwarded to the next level with a copy of the register? | (Yes/No) |  | |
| 7.14 | How long was the total vaccination session for all girls vaccinated | (Yes/No) | Time girls left class:  Time returned to class: | |
| **8** | **POST-VACCINATION** |  |  | |
| **8.1** | Are the girls observed for 15 minutes post vaccination? | (Yes/No) |  | |
| 8.2 | Were there any Adverse Events Following Immunisation reported | (Yes/No) |  | |
| 8.3 | Are unused vaccine and supplies appropriately packed for transportation back to HPV vaccine supply point | (Yes/No) |  | |
| 8.4 | Was the vaccine area left clean with no waste materials? | (Yes/No) |  | |
| 9 | Stock control |  |  | |
| 9.1 | Are all stock received for the HPV recorded on receipt? | (Yes/No) |  | |
| 9.2 | Is stock taken, used and returned reported daily? | (Yes/No) |  | |
| 9.3 | Are vaccine stocks recorded accurately? | (Yes/No) |  | |
| 9.4 | Are vaccine stocks checked daily and weekly | (Yes/No) |  | |
| 9.6 | Are vaccines that are spoilt (broken, frozen or cold chain compromised) reported and recorded accordingly | (Yes/No) |  | |
| **10** | **Data & Records** |  |  | |
| 10.1 | Are vaccinations recorded on vaccination cards and registers? | (Yes/No) |  | |
| 10.2 | Are consent forms properly checked and kept safely? | (Yes/No) |  | |
| 10.3 | Are weekly summary sheets compiled? | (Yes/No) |  | |
| 10.4 | Are weekly summary sheets submitted to facility manager/sub-district office/district? | (Yes/No) |  | |
| 10.5 | Is data captured weekly by Tuesday for the previous week? | (Yes/No) |  | |
| 10.6 | Are the captured data files for the previous week submitted to province by Wed? | (Yes/No) |  | |
| 10.7 | Is all the data national submitted every Thursday | (Yes/No) |  | |
